# Editorial: Microbial-driven carbon, nitrogen and phosphorus cycling mechanisms in terrestrial ecosystems

**DOI:** 10.3389/fmicb.2026.1858133

**Published:** 2026-05-07

**Authors:** Zhuo Hao, Lei Jiang, Congcong Liu, Yuqian Tang, Yang Yang

**Affiliations:** 1 Institute of Environment and Sustainable Development in Agriculture, Chinese Academy of Agricultural Sciences, Beijing, China; 2 State Key Laboratory for Development and Utilization of Forest Food Resources, Zhejiang A&F University, Hangzhou, China; 3 College of Life and Environmental Sciences, Minzu University of China, Beijing, China; 4 College of Agriculture and Biological Sciences, Dali University, Dali, Yunnan, China; 5 College of Forestry, Wildlife and Environment, Auburn University, Auburn, AL, United States

**Keywords:** biogeochemistry, critical zone, global environmental change, microbial communities, microbial metabolic pathways, nutrient acquisition strategy, stoichiometry

Microorganisms underpin the stability and multifunctionality of terrestrial ecosystems by driving the coupled biogeochemical cycles of carbon (C), nitrogen (N), and phosphorus (P). Distributed from deep subsurface mineral layers to surface soils, rhizosphere interfaces, plant phyllospheres, rivers, lakes, and wetlands, these organisms mediate organic matter decomposition, mineral weathering, nutrient transformation, and symbiotic resource exchange, thereby linking hydrological, geochemical, and biological processes to sustain primary productivity, soil fertility, and carbon sequestration. Under rapid global change, including climate warming, altered precipitation, land-use conversion, anthropogenic pollution, and soil degradation, however, microbial community assembly, functional traits, metabolic pathways, and interaction networks are being dramatically reshaped (Mukhtar et al., 2023; You et al., 2024). Although rapid advances in metagenomics, metabolomics, stable isotope probing, high-throughput quantitative PCR, and ecosystem modeling have deepened our understanding of microbial functional diversity, critical knowledge gaps remain in mechanistically linking microbial physiological processes to ecosystem-scale C-N-P dynamics and feedbacks to environmental perturbations (Fierer, 2017). This Research Topic was established to bridge these gaps, integrating microbial functional ecology, biogeochemistry, and ecosystem resilience to advance predictive understanding in a rapidly changing world.

A central theme emerging across this Research Topic is that microbial functional traits represent the fundamental mechanistic link between microbial physiology and ecosystem-level biogeochemical function. Analogous to plant functional traits that govern responses to environmental stress, microbial traits-including extracellular enzyme secretion, nutrient uptake kinetics, carbon use efficiency, and functional gene regulation-determine the rates, stoichiometric balance, and stability of C, N, and P transformations (Chen and Chen, 2021). These traits govern how microbial communities respond to resource availability, environmental filtering, and disturbance, and define whether ecosystems sustain tightly coupled nutrient cycling or shift toward inefficient, decoupled states (Moorhead et al., 2016). Across contrasting ecosystems including croplands, forests, saline-alkali soils, oil-contaminated sites, orchards, and wetlands, consistent patterns emerge: microbial investment in nutrient-acquiring enzymes shifts predictably under resource limitation; functional gene expression reflects selective environmental filtering; and community assembly reflects evolutionary trade-offs between nutrient cycling capacity and stress tolerance. Supported by high-resolution molecular and statistical evidence, this Research Topic consolidates a trait-based framework that unifies understanding of microbial contributions to terrestrial biogeochemical cycling (Trivedi et al., 2021).

Equally robust insights emerge from the integration of microbial functional ecology with ecological stoichiometry and environmental stress responses. Similar to plant stoichiometric homeostasis, microbial communities adjust metabolic pathways and functional gene expression to maintain stoichiometric balance under fluctuating C-N-P supply (Elser et al., 2000; Yuan et al., 2025). Under drought, salinity, hydrocarbon contamination, or nutrient imbalance, microbes prioritize stress tolerance or nutrient scavenging strategies, often reducing biogeochemical efficiency and slowing element turnover (El-Beltagi et al., 2026). Long-term mineral fertilization, monoculture planting, oil pollution, and soil salinization disrupt stoichiometric equilibrium, reduce functional diversity, and suppress keystone functional groups such as *phoD*-harboring bacteria that regulate organic phosphorus mineralization (Ragot et al., 2015). By contrast, sustainable management interventions-including balanced organic-mineral fertilization, conversion to native tree species, integrated rhizosphere and phyllosphere microbial inoculation, and electroactive microbial enhancement-restore stoichiometric coupling, strengthen cooperative microbial networks, and enhance multifunctionality. These findings collectively establish microbial stoichiometry and functional traits as sensitive and reliable indicators of ecosystem resistance and resilience under global change.

A further conceptual advance of this Research Topic is the expansion of microbial functional ecology into underrepresented ecosystems and emerging global-change drivers. The compiled studies address previously understudied systems and stressors, including petroleum-contaminated soils, saline-alkali agricultural lands, subtropical plantation conversion, orchard phyllosphere-rhizosphere interactions, and soil bioelectronic systems mediated by electroactive microorganisms. Collectively, these contributions reveal that microbial functional responses are ecosystem-specific, soil-depth-dependent, and strongly modulated by management practices. For example, converting Eucalyptus monocultures to native tree plantations alleviates microbial carbon limitation but intensifies phosphorus limitation, particularly in surface soils, while enhancing overall enzyme-mediated nutrient mobilization in subsoils. In saline-alkali soils, a 50% organic-50% mineral fertilization regime significantly reduces pH and salinity, enriches beneficial microbial taxa, and upregulates functional genes involved in C fixation (*acsA, mct*), N fixation (*nifH*), and P mobilization (*phoD, ppx*) while reducing nitrification and denitrification pathways associated with N loss (Wei et al., 2021). In oil-contaminated soils, pollution reduces *phoD*-harboring bacterial abundance, diversity, and alkaline phosphatase activity, exacerbating P limitation and inhibiting hydrocarbon biodegradation. In orchard systems, combined soil and foliar microbial inoculants restructure bacterial networks, increase beneficial phyla such as *Actinomycetota* and *Bacillota*, and significantly enhance yield and fruit quality. Meanwhile, electroactive microorganisms extend biogeochemical cycling through extracellular electron transfer, enabling self-regulated nutrient cycling, pollutant remediation, and carbon sequestration in bioelectronic soil systems (Logan, 2009). Together, these findings broaden the empirical and theoretical boundaries of microbial biogeochemistry and support robust, context-aware predictions under world global change.

Based on the synthetic insights from this Research Topic, we highlight high-priority directions for future research. First, more integrative conceptual and quantitative models are urgently needed to explicitly link microbial physiological processes, functional traits, and community assembly rules to ecosystem C-N-P dynamics. Second, future studies must better address the interactive effects of multiple co-occurring stressors-including climate change, salinization, drought, pollution, and land-use change-on microbial functional stability. Third, trait-based microbial indicators should be developed and validated for monitoring soil health, nutrient limitation, and restoration effectiveness across biomes and management regimes. Fourth, greater research attention must be devoted to vertical stratification, deep-soil microbial processes, plant-microbe-soil feedbacks, and electroactive microbial mechanisms that underpin next-generation regenerative agriculture and bioremediation.

In summary, this Research Topic establishes microbial-driven C-N-P cycling as a central pillar of terrestrial ecosystem functional ecology. By integrating microbial physiology, functional traits, community dynamics, and biogeochemical processes, the compiled studies clarify how microorganisms regulate ecosystem function and respond to global environmental change. The trait-based, stoichiometric, and network-oriented perspectives presented here provide new quantitative and conceptual tools for predicting ecosystem responses, restoring degraded lands, improving sustainable agricultural production, and enhancing climate resilience. We anticipate these insights will guide future theoretical developments, experiments, cross-ecosystem syntheses, and applied land-management strategies.

The case studies, hypothesis and theory articles published in this Research Topic demonstrates that microbial functional ecology is indispensable to understanding, managing, and sustaining terrestrial biogeochemical cycles in the Anthropocene. We express sincere gratitude to all contributing authors, expert reviewers, and editorial support staff for making this interdisciplinary Research Topic possible. We hope this volume stimulates continued scientific advancement toward a more mechanistic, predictive, and applicable science of microbial-driven ecosystem function, supporting global efforts toward ecosystem restoration, food security, and climate stability.
